# Measured depth of subcutaneous tissue on posterolateral arm of aeroallergen immunotherapy patients

**DOI:** 10.1186/1710-1492-8-S1-A7

**Published:** 2012-11-02

**Authors:** Laura Kim, Ryan Potts, Clark Eeuwes, Arunmozhi Dominic, Immaculate Nevis, Harold L Kim

**Affiliations:** 1 McGill University, Montréal, Québec, Canada; 2 University of Waterloo, Waterloo, Ontario, Canada; 3 University of Western Ontario, London, Ontario, Canada; 4 McMaster University, Hamilton, Ontario, Canada

## Background

Subcutaneous immunotherapy (SIT) injections for aeroallergens are often utilized in patients. SIT should be injected into the subcutaneous space in the mid-posterolateral upper arm. If the injections are given intramuscularly (IM), there may be an increased risk of anaphylaxis. In our allergy clinic, SIT is given with *BD Safety Glide™ Allergy* syringes with needle length 13 mm. There is a risk of the SIT being injected IM if patients have a skin to muscle depth (STMD) less than 13 mm.

## Methods

Charts were reviewed in an allergy clinic for patients on SIT where an ultrasound of the left posterolateral arm was done to measure STMD. Baseline characteristics of the two groups of patients with STMD greater than, and less than or equal to 13 mm were compared. The proportions of patients with STMD greater than 4mm, 6 mm, 8 mm, and 10 mm were calculated. Multivariable logistic regression was performed with age, sex, BMI and race.

## Results

Ultrasounds had been completed on 186 patients on SIT. There were 149 (80%) with STMD less than 13mm. Baseline characteristics including age, sex and BMI differed among the two groups (*p* < 0.05). Based on the logistic regression analysis, BMI was significantly associated with STMD. There were 168 (90%) patients with more than 4mm STMD.

## Conclusions

With standard allergy syringes, most patients on SIT are at risk of receiving the injections IM. A needle length of 4 mm would significantly decrease the risk of SIT being given IM.

